# 急性髓系白血病中CEBPA基因突变的类型及其对预后的影响

**DOI:** 10.3760/cma.j.cn121090-20240316-00098

**Published:** 2024-06

**Authors:** 玥莹 毛, 昊 蔡, 欣欣 曹, 俊 冯, 路 张, 道斌 周, 剑 李

**Affiliations:** 中国医学科学院、北京协和医学院北京协和医院血液内科，北京 100730 Department of Hematology, Peking Union Medical College Hospital, Chinese Academy of Medical Sciences & Peking Union Medical College, Beijing 100730, China

**Keywords:** 白血病，髓系，急性, CEBPA基因突变, 临床特点, 预后, Leukemia, myeloid, acute, CEBPA gene mutation, Clinical characteristics, Prognosis

## Abstract

**目的:**

探讨携带CEBPA基因突变的急性髓系白血病（AML）患者的突变类型、临床特点和突变对生存结局的影响。

**方法:**

回顾性分析2016年4月至2022年11月期间北京协和医院确诊的57例伴有CEBPA基因突变的AML患者的人口学信息、临床表现、实验室检查结果、治疗以及生存数据。

**结果:**

57例CEBPA基因突变患者占同期所有353例AML患者的16.1％，其中bZIP区域框内突变（CEBPA-bZIPinf）28例，其余CEBPA基因突变（CEBPA-other）29例。与CEBPA-other患者相比，CEBPA-bZIPinf患者更年轻（54岁对64岁，*P*＝0.010），原发性AML更常见（*P*＝0.001），骨髓原始细胞比例更高（68.0％对36.3％，*P*＝0.001）。CEBPA-bZIPinf及CEBPA-other患者分别有24例和19例接受化疗，CEBPA-bZIPinf患者的1个疗程完全缓解率显著高于CEBPA-other（87.5％对47.4％，*P*＝0.010）及CEBPA野生型（87.5％对50.3％，*P*＝0.002）患者。中位随访11个月，CEBPA-bZIPinf患者的中位总生存期明显长于CEBPA野生型患者（未达到对22.1个月，*P*＝0.012）。

**结论:**

CEBPA-bZIPinf突变的AML患者具有独特的临床特征，对化疗的反应更好，预后更佳。

## Abstract (en)

**Objective:**

To demonstrate the type of CEBPA gene mutations among patients with acute myeloid leukemia（AML）, clinical characteristics, and prognostic effect on patient outcomes.

**Methods:**

Demographic data, clinical features, laboratory characteristics, and data about treatment and follow-up of 57 patients with CEBPA mutated AML diagnosed at Peking Union Medical College Hospital between April 2016 and November 2022 were collected and analyzed.

**Results:**

In total, 57 patients with CEBPA mutation accounted for 16.1％ of all the 353 patients with AML, among which 28 patients had CEBPA-bZIPinf and 29 had CEBPA-other. Compared with the CEBPA-other group, the CEBPA-bZIPinf group was younger（54 *vs* 64 years, *P*＝0.010）, de novo AML was more common（*P*＝0.001）, and the level of bone marrow blast was higher（68.0％ *vs* 36.3％, *P*＝0.001）. Moreover, 24 patients from the CEBPA-bZIPinf group and 19 from the CEBPA-other group received chemotherapy. The one-course complete remission（CR）rate of the CEBPA-bZIPinf group was significantly higher than that of the CEBPA-other（87.5％ *vs* 47.4％, *P*＝0.010）and CEBPA-wt（87.5％ *vs* 50.3％, *P*＝0.002）groups. After a median follow-up of 11 months, the median OS of the CEBPA-bZIPinf group was significantly longer than that of the CEBPA-wt group（not reached *vs* 22.1 months, *P*＝0.012）.

**Conclusion:**

CEBPA-bZIPinf mutated AML is a unique clinical entity, with a younger age of diagnosis, better response to chemotherapy, and better prognosis.

CCAAT-增强子结合蛋白α（CEBPA）基因定位于染色体19q3，是转录因子碱性亮氨酸拉链（basic leucine zipper，bZIP）家族的成员。CEBPA基因的N末端区域有用于转录控制的反向激活结构域（TAD1和TAD2），而C末端为bZIP结构域。该基因主要在髓系细胞中表达，在维持早期造血阶段髓系细胞增殖与分化的平衡中起到重要作用[1]–[2]。急性髓系白血病（AML）患者的CEBPA基因突变发生率为4％～20％[3]。CEBPA基因突变对AML的预后价值在2002年被首次描述[4]–[5]，此后WHO 2016版分类标准和ELN 2017版成人AML诊治指南均提出CEBPA双等位基因突变为预后良好因素[6]–[7]。然而，近年来多项研究表明，CEBPA基因突变带来的良好预后价值取决于突变位置是否位于bZIP结构域，特别是bZIP域的框内突变，而不取决于突变数目为单突变还是双突变[3],[8]–[9]。ELN 2022版指南也随之将预后参考指标由CEBPA双突变调整为CEBPA框内bZIP突变[10]。目前国内仍鲜有关于AML患者CEBPA基因突变的系列报道。故本研究拟回顾性分析我院确诊的AML患者CEBPA基因突变情况，并验证不同突变类型对预后的影响。

## 病例与方法

1. 患者资料：本研究纳入了2006年3月至2022年11月，就诊于北京协和医院并明确诊断为非急性早幼粒细胞白血病（APL）的所有AML患者。所有患者的诊断根据2016版WHO白血病分类标准[6]确立。

2. 细胞遗传学、分子生物学分析：采集患者骨髓（骨髓无法获得者使用外周血），进行细胞遗传学和分子生物学分析。采用R显带方法进行染色体核型分析。突变分析采用涵盖42个基因的二代测序平台完成[11]。每个样本的平均深度为2 500×，平均5％的目标序列被充分深度覆盖以识别变异。

3. 主要观察指标及随访：通过查阅门诊、住院病历及电话进行随访，末次随访时间为2023年2月。记录患者的1个疗程诱导化疗后的完全缓解（CR）率及总生存（OS）时间。CR判断参照2017年ELN缓解标准[7]。OS时间为确诊至患者死亡或末次随访时间（失访时间）。

4. 统计学处理：采用SPSS 26进行统计学分析。连续变量的组间差异采用Mann-Whitney *U*非参数检验，率的比较采用Fisher精确检验；生存分析采用Kaplan-Meier法，并用Log-rank检验进行单因素分析。所有统计学分析均为双侧检验，*P*<0.05为差异有统计学意义。采用GraphPad 9.4.0进行生存曲线绘制。

## 结果

1. CEBPA突变状态：2016年4月至2022年11月，我院新诊断的非APL的AML患者共353例，其中携带CEBPA基因突变者共57例（16.1％）。在所有CEBPA基因突变的AML患者中，单等位基因突变（CEBPAsm）患者12例（21.1％），双等位基因突变（CEBPAdm）患者44例（77.2％），另有1例（1.8％）携带3处CEBPA突变（CEBPAtm）。CEBPA突变位置和类型方面，CEBPAsm患者中有5例（41.7％）位于bZIP区域（CEBPAsm-bZIP），其中框内突变（CEBPAsm-bZIPinf）1例。CEBPAdm患者中有38例（86.4％）存在位于bZIP区域（CEBPAdm-bZIP）的突变，其中3例（6.8％）两个突变均位于bZIP区域，35例（79.5％）有一个突变位于bZIP区域，6例（13.6％）两个突变均在bZIP区域之外；含bZIP区域突变的患者中，框内突变（CEBPAdm-bZIPinf）27例。1例CEBPAtm患者3处突变均不在bZIP区域之内。将含有CEBPA-bZIP框内突变的所有患者统一归类为CEBPA-bZIPinf，其余CEBPA基因突变患者归类为CEBPA-other。

2. CEBPA基因突变患者的临床特点：表1比较了CEBPA基因不同突变状态与同期CEBPA野生型（CEBPA-wt）患者的临床与实验室特点。CEBPA-bZIPinf患者的诊断年龄小于CEBPA-other患者（54岁对64岁，*P*＝0.010），但与CEBPA-wt患者相近。全部28例CEBPA-bZIPinf患者均为原发性AML，无其他髓系疾病转化或治疗相关的AML，与CEBPA-other（*P*＝0.001）、CEBPA-wt（*P*＝0.016）患者均存在明显差异。CEBPA-bZIPinf患者的骨髓原始细胞数量明显高于CEBPA-other患者（68.0％对36.3％，*P*＝0.001），也高于CEBPA-wt患者。

**表1:** CEBPA不同突变状态急性髓系白血病（AML）患者的临床特征比较

临床特征	CEBPA-bZIPinf（28例）	CEBPA-other（29例）	CEBPA-wt（296例）	*P*_1_值	*P*_2_值
诊断年龄[岁，*M*（范围）]	54（24~74）	64（28~85）	53（14~88）	0.010	0.502
性别（男女比值）	1.3	1.6	1.3	0.790	1.000
AML类型（例）					
原发性	28	19	233	0.001	0.016
非原发性（髓系疾病转化、治疗相关）	0	10	63		
骨髓原始细胞[%，*M*（范围）]	68.0（20.5~80.0）	36.3（20.5~83.5）	50.5（0~98.0）	0.001	0.002
治疗前WBC[×10^9^/L，*M*（范围）]	11.57（2.03~297.75）	7.11（1.50~240.20）	8.48（0.36~362.56）	0.239	0.080

**注** CEBPA-bZIPinf：含CEBPA基因bZIP区域框内突变；CEBPA-other：其余CEBPA基因突变；CEBPA-wt：CEBPA基因野生型；*P*_1_值：CEBPA-bZIPinf组与CEBPA-other组比较；*P*_2_值：CEBPA-bZIPinf组与CEBPA-wt组比较

3. CEBPA基因突变患者的分子和细胞遗传学特点：图1描述了CEBPA基因不同突变状态AML患者的伴随突变和细胞遗传学特点。CEBPA-bZIPinf患者伴随其他突变较少［中位1（0～2）个］，而CEBPA-other患者伴随其他突变较多［中位2（0～5）个］。CEBPA-bZIPinf患者伴随的高频突变（≥10％）包括WT1（25.0％）、TET2（17.9％）、NRAS（14.3％）、GATA2（10.7％）；而CEBPA-other患者伴随的高频突变包括TET2（27.6％）、IDH2（17.2％）、STAG2（17.2％）、WT1（13.8％）、FLT3（13.8％）、SRSF2（13.8％）、NRAS（10.3％）、NPM1（10.3％）、DNMT3A（10.3％）、ASXL1（10.3％）、CSF3R（10.3％）。

**图1:**
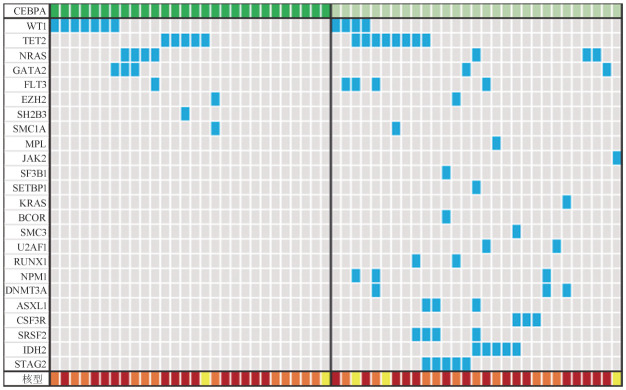
携带CEBPA基因突变急性髓系白血病患者分子和细胞遗传学特点 **注** 深绿色：含CEBPA基因bZIP区域框内突变；浅绿色：其余CEBPA基因突变；蓝色：突变；灰色：野生型；红色：正常核型；橙色：异常核型；黄色：核型未知

4. 治疗情况及生存分析：28例CEBPA-bZIPinf患者中有24例接受化疗，其中18例接受标准（“3+7”方案，柔红霉素≥60 mg·m^−2^·d^−1^或去甲氧柔红霉素≥12 mg·m^−2^·d^−1^第1～3天；阿糖胞苷100 mg·m^−2^·12 h^−1^第1～7天）化疗，6例接受低强度化疗；1个疗程诱导化疗后，21例（87.5％）患者达到CR。29例CEBPA-other患者中有19例接受化疗，其中8例接受标准化疗，11例接受低强度化疗；1个疗程诱导化疗后，9例（47.4％）患者达到CR。而296例CEBPA-wt患者中，294例接受化疗，其中180例接受标准化疗，114例接受低强度化疗；1个疗程诱导化疗后，148例（50.3％）患者达到CR。CEBPA-bZIPinf患者1个疗程化疗后CR率显著高于CEBPA-other（*P*＝0.010）、CEBPA-wt患者（*P*＝0.002）。接受强化疗的206例患者中，CEBPA-bZIPinf组18例患者全部CR，CEBPA-other组8例患者CR率75％，CEBPA-wt组180例患者CR率64％，差异有统计学意义（*P*＝0.021）。所有存活患者均接受巩固治疗，CEBPA-bZIPinf、CEBPA-wt患者分别有4例、40例接受异基因造血干细胞移植，CEBPA-other患者均未移植。对接受化疗的所有患者进行随访，截至2023年2月，中位随访时间11（0～73）个月，141例患者已经死亡，其中CEBPA-bZIPinf患者4例（16.7％）死亡，CEBPA-other患者5例（26.3％）死亡。CEBPA-bZIPinf与CEBPA-other患者的中位OS期均尚未达到，CEBPA-wt患者中位OS期22.1个月，CEBPA-bZIPinf患者生存优于CEBPA-wt患者（*HR*＝0.287，95％*CI* 0.106～0.776）（图2）。

**图2:**
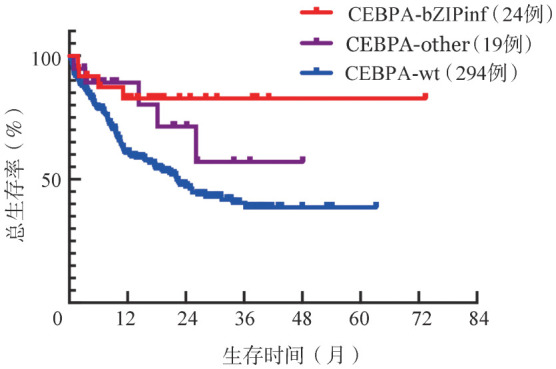
CEBPA-bZIPinf、CEBPA-other与CEBPA-wt急性髓系白血病患者的生存曲线 **注** CEBPA-bZIPinf：含CEBPA基因bZIP区域框内突变；CEBPA-other：其余CEBPA基因突变；CEBPA-wt：CEBPA基因野生型

## 讨论

自2021年开始，AML患者中的CEBPA-bZIP突变开始逐渐引起关注。我们AML队列中CEBPA基因突变率为16.1％，与以往文献报道的相似。而CEBPA-bZIPinf突变的发生率在各团队报道中差异较大，在17.5％～81％不等[2]，原因可能与各研究纳入的不同人群（如儿童、原发AML、正常核型患者等）有关。我们研究的队列为本中心真实世界的回顾性连续队列，包含异常核型、老年、继发性AML、治疗相关AML等各类AML患者，CEBPA基因突变患者中CEBPA-bZIPinf突变发生率为49.1％。

以往文献显示，CEBPA-bZIPinf患者相较于CEBPA-other和CEBPA-wt患者群体，是相对独立的一个整体，有较为特征性的临床表现。他们起病时更为年轻[3],[12]，外周血WBC水平更高[3]。我们的研究同样有类似的发现。以往文献也提出CEBPA-bZIPinf患者有较为一致的伴随突变，其中多篇文献提到GATA2突变发生率较高，部分文献报道高达30％以上[3],[12]–[13]。而在我们的研究中，发现CEBPA-bZIPinf比CEBPA-other患者的突变数目更少，而在高频突变及染色体核型方面均未发现明显的差异。

治疗方面，我们队列中CEBPA-bZIPinf组接受标准化疗的患者比例远高于CEBPA-other和CEBPA-wt组，可能由于这组患者相对年轻，且均为原发性AML，更有希望从强化疗中获益。同时CEBPA-bZIPinf组患者的CR率也远高于其余两组。在均接受强化疗的患者中，CEBPA-bZIPinf组的缓解率也远高于另外两组，支持良好的治疗反应不仅仅由化疗强度决定，也与疾病本身的特点密切相关。与CR率类似，CEBPA-bZIPinf患者的OS也明显好于CEBPA-other及CEBPA-wt患者，与文献报道相一致[3],[8]–[9],[13]。

本研究队列中CEBPAsm仅有1例患者携带CEBPA-bZIPinf突变，该患者诊断年龄53岁，骨髓原始细胞比例66.5％，经标准化疗后达到CR，符合CEBPA-bZIPinf组的一般特点。但仍因本组数量较少，可能使研究结果产生一定偏倚。此外，本研究队列为真实世界队列，受限于此，CEBPA双突变患者未进行胚系突变分析，且未对患者的治疗方案进行干预设计，均可能对预后分析产生一定影响。

综上所述，CEBPA-bZIPinf突变的AML患者是一个相对独立的临床整体，此类患者发病年龄较轻，以原发性AML最常见，骨髓原始细胞比CEBPA-other患者更多，伴随突变更少，预后比CEBPA-other及CEBPA-wt患者更好。
